# Metabolomic Analysis of Flavour Development in Mung Bean Foods: Impact of Thermal Processing and Storage on Precursor and Volatile Compounds

**DOI:** 10.3390/foods14050797

**Published:** 2025-02-26

**Authors:** Jingru Sun, Yanlong Li, Xiaoyu Cheng, Hongli Zhang, Jinchi Yu, Lixiang Zhang, Ying Qiu, Jingjing Diao, Changyuan Wang

**Affiliations:** 1 College of Food Science, Heilongjiang Bayi Agricultural University, Daqing 163319, China; s15545723965@163.com (J.S.); a99008191@163.com (Y.L.); 18845620738@163.com (X.C.); 15563984489@163.com (H.Z.); y02j11c20@126.com (J.Y.); 15067512982@163.com (L.Z.); 13504508671@139.com (Y.Q.); diaojing62@163.com (J.D.); 2 Department of National Coarse Cereals Engineering Research Center, Heilongjiang Bayi Agricultural University, Daqing 163319, China

**Keywords:** mung bean, hot processing, storage, metabolomics, flavours

## Abstract

Consumers prefer mung beans for their low allergenicity and nutritional benefits. However, flavour development in mung bean foods has been problematic, with beany flavour being a limiting factor. Hot processing is crucial in forming mung bean flavours, and storage-induced changes in flavour precursors directly impact the taste post-processing. This study used metabolomics to analyse the effects of hot processing (baking and cooking) on mung bean flavour and differences after storage. A total of 131 flavour precursors and 45 volatile substances were identified across six sample groups. The results showed that baking and cooking upregulated 22 and 18 volatile substances (ketones, aldehydes, esters, pyridine, pyrazines, etc.), respectively. The Maillard reaction during baking notably increased compounds like 2-hydroxypyridine, 2-methoxy-3-isobutyl pyrazine, 1,2-hexanedione, and 2,3-butanedione. Both methods inhibited linoleic acid oxidation, significantly reducing hexanal content, a key “bean” odour substance. However, storage accelerated linoleic acid conversion to C13 peroxides, increasing hexanal content and bean odour. This process decreased precursor substances like glucose-1-phosphate and caused the accumulation of pyruvic acid intermediates in pentose phosphate and pyruvate metabolism/amino acid metabolism pathways, leading to reduced mung bean taste richness.

## 1. Introduction

Mung beans are among the most significant leguminous plants in Asia and are widely valued for their nutritional and medicinal properties. Mung beans contain various bioactive compounds, including resistant starch, polysaccharides, and flavonoids, which have been shown to possess multiple health benefits, such as the ability to lower lipids, act as antioxidants, and prevent tumour growth. Besides their nutritional value, active substances of mung beans are recognised for their ability to strengthen antioxidant, anti-inflammatory, and anti-heat stress responses [1]. Notwithstanding their substantial nutritional and medicinal value, the “beany flavour” of mung beans poses a significant challenge in terms of their application and promotion. This flavour is often perceived as unpleasant by numerous people and negatively affects the production and market acceptance of mung bean products, especially by a broader range of consumers. Research has identified aldehydes and ketones as the primary compounds responsible for this “beany flavour”, which directly affects consumer acceptance [2]. Consequently, enhancing the flavour of mung beans has emerged as a pivotal challenge for advancing deep processing and broader promotion.

Thermal processing is a critical process in the food industry. Thermal treatments, such as microwaving, roasting, steaming, and frying, generate flavour compounds, resulting in changes in sensory characteristics. Appropriate thermal processing conditions have been shown to enhance the taste and aroma of products, increasing their appeal and stimulating consumer appetite. Lasekan et al. [3] and Lemarcq et al. [4] found that the aroma contributions of foods subjected to roasting and steaming primarily depend on volatile compounds, such as aldehydes, ketones, pyrazines, and alcohols. These flavours and aromas are derived from the reduction, degradation, oxidation, condensation, and rearrangement of nutrients in food, such as proteins, carbohydrates, and fats. These processes catalyse the formation of flavour compounds [5,6]. Consequently, it is imperative to acknowledge the impact of processing on the flavour precursors. In addition, the processing method has been shown to substantially influence the formation of flavour compounds. As the degree of thermal treatment increases, volatile compounds accumulate with prolonged heat exposure [7]. In addition, Elmore et al. [8] discovered that when aldehydes are present in high proportions, they can further react with the decomposition products of the Maillard reaction, ultimately resulting in an undesirable off-flavour.

Steaming has been shown to effectively remove the raw beany flavour of mung beans, making their texture soft and sticky, and enhancing their palatability [9]. During high-heat processing, the Maillard reaction [10] imparts unique aroma and flavour characteristics to mung beans [11]. However, the chemical compounds that contribute to the flavour quality of mung beans, chemical composition of the characteristic flavour compounds in traditionally heat-processed mung beans, and impact of different processing methods on the generation of key volatile flavour compounds remain to be elucidated. During legume storage, changes in moisture, proteins, carbohydrates, fats, and endogenous metabolites significantly influence the formation and transformation of flavour compounds, altering the final flavour quality of legumes [12,13]. Nevertheless, current research on the changes in nutritional components, flavour precursors, and flavour compounds of mung beans during storage remains insufficient. In addition, there is a paucity of research addressing whether the flavour quality of processed products can be maintained after storage.

This study investigated the effects of heat processing on fresh mung beans and mung beans stored for one year (at 20 °C, 12.5% humidity, and protected from light). Two processing methods were applied: roasting at 150 °C for 1 h and boiling at 100 °C for 30 min. Using GC-MS technology, the changes in volatile flavour compounds in mung beans before and after storage were analysed. The first objective of this study was to identify key flavour metabolites responsible for differences caused by storage and to elucidate the associated metabolic pathways. The second objective was to explore the effects of different processing methods on the generation of flavour compounds in mung beans, as well as the changes in flavour compounds and precursors after storage. Ultimately, this study aimed to uncover the mechanisms underlying the formation of key volatile flavour compounds in processed mung beans. The findings provide a theoretical basis for optimising mung bean processing methods and enhancing their nutritional value, as well as offering valuable insights for the development of mung bean processing technologies and related products.

## 2. Materials and Methods

### 2.1. Materials

Mung beans were obtained in the fall of 2023 from Datong City, Shanxi Province, China. The variety of mung beans was Green Mung Bean. Mung bean samples with intact beans and bright colours were selected and stored at −80 °C.

### 2.2. Reagents and Instrumentation

Anhydrous ethanol (Micklin Biochemical Technology Co., Ltd., Shanghai, China), petroleum ether (Micklin Biochemical Technology Co., Ltd., Shanghai, China), potassium hydroxide (Micklin Biochemical Technology Co., Ltd., Shanghai, China), and potassium iodide (Micklin Biochemical Technology Co., Ltd., Shanghai, China) were purchased. Hydrochloric acid (Sinopharm Chemical Reagent Co., Ltd., Shanghai, China) and sodium hydroxide (Sinopharm Chemical Reagent Co., Ltd., Shanghai, China) were purchased. Chloroform (Adamas Reagent Co., Ltd., Shanghai, China) and pyridine (Adamas Reagent Co., Ltd., Shanghai, China) were purchased. High temperature-tolerant α-amylase (Shanghai Aladdin Biochemical Technology Co., Ltd., Shanghai, China) was obtained. Methanol (CNW Technologies GmbH, Düsseldorf, Germany) was obtained. Methoxyamine hydrochloride (Tokyo Chemical Industry Co., Ltd., Tokyo, Japan) was purchased. L-2-chlorophenylalanine (Shanghai Hengbo Biotechnology Co., Ltd., Shanghai, China) was purchased. BSTFA (REGIS Technologies, Inc., Morton Grove, IL, USA) was obtained. Saturated fatty acid methyl esters (Dr. Ehrenstorfer GmbH, Augsburg, Germany) were purchased.

TG16-WS centrifuge (Changsha Xiangyi Centrifuge Instrument Co., Ltd., Changsha, Hunan, China) was obtained. DELTA 320 pH meter (Mettler-Toledo Instrument Co., Ltd., Shanghai, China) and HHS-21-6 digital constant-temperature water bath (Shanghai Jinghong Laboratory Instrument Co., Ltd., Shanghai, China) were sourced. A360 UV-visible spectrophotometer (Shanghai Aoyi Technology Co., Ltd., Shanghai, China) was purchased. DGG-9140 electric constant-temperature blast-drying oven (Shanghai Bosheng Laboratory Equipment Co., Ltd., Shanghai, China) was obtained. CHA-2A constant-temperature oscillator (Changzhou Shenguang Instrument Co., Ltd., Changzhou, Jiangsu, China) was obtained. 7890 gas chromatography system (Agilent Technologies, Santa Clara, CA, USA) and DB-5MS chromatographic column (30 m × 250 μm × 0.25 μm, Agilent Technologies, Santa Clara, CA, USA) were purchased.

### 2.3. Sample Processing

#### 2.3.1. Fresh Mung Beans

The freshly picked mung beans were washed with deionised water and dried at room temperature to obtain fresh mung beans. The dried samples were ground in a grinder and passed through a 70-mesh sieve to obtain a fresh mung bean powder. In the following, M was used instead of fresh mung beans.

#### 2.3.2. Storage Mung Beans

The freshly picked mung beans were stored in dry, dark, and room-temperature conditions for one year, washed with deionized water, dried, and ground, and passed through a 70-mesh sieve to obtain storage mung bean powder. In the following, SM was used instead of storage mung beans.

#### 2.3.3. Cooking Process

The mung beans were washed with deionised water, dried according to the steps described by Xu et al. [14], weighed, and soaked 16 h at room temperature (25 °C) to ensure a hydration rate of 50%. The soaked samples were placed in a rice cooker with a material-to-water ratio of 1:500. After cooking for 40 min, the beans were removed and oven-dried (60 °C) until they reached a constant weight. They were then ground in a grinder and passed through a 70-mesh sieve to obtain cooked mung bean powder. In the following, MC and SMC were used instead of cooking mung beans and cooking mung beans after storage, respectively.

#### 2.3.4. Baking Process

The mung beans were washed with deionised water and baked in an oven at 150 °C for 1 h. After removing the beans, they were crushed in a grinder and passed through a 70-mesh sieve to obtain roasted mung bean powder. In the following, MB and SMB were used instead of baking mung beans and baking mung beans after storage, respectively.

### 2.4. Total Amino Acids (TAA) Content

The TAA content of M, SM, MC, MB, SMC, and SMB was determined using an amino acid analyser. Refer to GB/T5009.124-2016 [15] for the determination methods. An appropriate amount of sample was added to 6 mol/L HCl, hydrolysed at high temperature for 22 h, and then the pH was adjusted to 2.2 with citric acid buffer after cooling. The solution was filtered through 0.22-μm membrane and then detected using a machine [16].

### 2.5. Gas Chromatography-Mass Spectrometry

#### 2.5.1. Preparation of Samples for Measurement

A sample of 50 ± 1 mg was placed in a 2 mL EP tube, and 500 μL of precooled extraction solution (methanol/water ratio = 3:1, containing the internal standard L-2-chlorophenylalanine) was added. The mixture was vortexed for 30 s. Steel beads were added, and the sample was processed with a 40 Hz grinder for 4 min, followed by ultrasound in an ice–water bath for 5 min (repeated three times). The sample was then centrifuged at 4 °C for 15 min at 12,000 rpm (13,800× *g*; radius, 8.6 cm). A volume of 200 μL of the supernatant was transferred to a 1.5 mL EP tube, and 200 μL of each sample was mixed to create a QC sample. The extract was then dried using a vacuum concentrator. After drying, 60 μL methoxyamine hydrochloride reagent (20 mg/mL dissolved in pyridine) was added to the dried metabolites, gently mixed, and incubated in an oven at 80 °C for 30 min. Next, 80 μL of BSTFA (containing 1% TMCS, *v*/*v*) was added to each sample, and the mixture was incubated at 70 °C for 1.5 h. After cooling to room temperature, 5 μL of FAMEs (dissolved in chloroform) was added to the mixed samples. The samples were then analysed in random order.

#### 2.5.2. Detection Conditions

An Agilent 7890 Gas Chromatography-Time-of-Flight Mass Spectrometry (GC-TOFMS) system was used, equipped with an Agilent DB-5MS capillary column (30 m × 250 μm × 0.25 μm, J&W Scientific, Folsom, CA, USA), and the sample was injected with a volume of 1 μL. The mode was set to the Splitless Mode, and the purge flow rate was 3 mL min^−1^. Helium was used as the carrier gas at a column flow rate of 1 mL min^−1^. The column temperature program was as follows: 50 °C for 1 min, raised to 310 °C at 10 °C min^−1^, and held for 8 min. The inlet, transfer line, and ion source temperatures were set at 280 °C, 280 °C, and 250 °C, respectively. The ionisation voltage was −70 eV, with a mass range of *m*/*z*: 50–500 and a scanning rate of 12.5 spectra per second. The solvent delay was set at 6.4 min.

### 2.6. Data Processing

#### 2.6.1. Data Preprocessing

Mass spectrometry data were processed using ChromaTOF software (V 4.3×, LECO) for peak extraction, baseline correction, deconvolution, integration, and alignment [17]. For the qualitative analysis of the LECO-Fiehn Rtx5 database, mass spectrum matching and retention time index matching were used. Finally, peaks with detection rates below 50% or RSD > 30% in QC samples were removed [18].

#### 2.6.2. Principal Component Analysis (PCA) and Orthogonal Partial Least Squares-Discriminant Analysis (OPLS-DA)

The data were first formatted in a centralised format using SIMCA software (V16.0.2, Sartorius Stedim Data Analytics AB, Umea, Sweden), followed by automatic modelling analysis. Subsequently, logarithmic transformation and parat-format processing were applied to the data using the SIMCA software. The first principal component was then subjected to OPLS-DA modelling, which included 7-fold cross-validation. R^2^ Y (the model’s explanatory power for categorical variable Y) and Q^2^ (the model’s predictive ability) obtained from cross-validation were used to evaluate the model’s effectiveness. Finally, the reliability of the injection model was validated through permutation testing, which involved randomly altering the order of categorical variable Y to obtain different random Q^2^ values.

#### 2.6.3. Screening of Differential Metabolites and Heatmap Maps

In this experiment, the following criteria were established to screen for differential metabolites: the *p*-value from Student’s *t*-test was less than 0.05, and the variable importance in projection (VIP) of the first principal component of the OPLS-DA model was greater than 1. The identified metabolites were visualised using a heatmap.

#### 2.6.4. Hierarchical Clustering Analysis of Differential Metabolites

By comparing the differential metabolites among groups, the Euclidean Distance Matrix (EDM) was calculated using their quantitative values. The differential metabolites were then clustered using the complete linkage method, and the results were presented as a heatmap.

#### 2.6.5. Metabolic Pathway Analysis of Differential Metabolites

Differentially expressed metabolites were subjected to pathway identification using the Kyoto Encyclopedia of Genes and Genomes (KEGG) pathway database. Functional information from genes and genomes serves as a clue to associating potential metabolic pathways with corresponding regulatory proteins, visually illustrating physiological and biochemical processes within the cell. The annotation of differential metabolites relied on KEGG and other authoritative databases, such as PubChem. After obtaining matching information for the differential metabolites, further searches were conducted on the pathway databases of the respective species to perform metabolic pathway analysis.

## 3. Results

### 3.1. Identification and Classification of Metabolites

**Figure 1:**
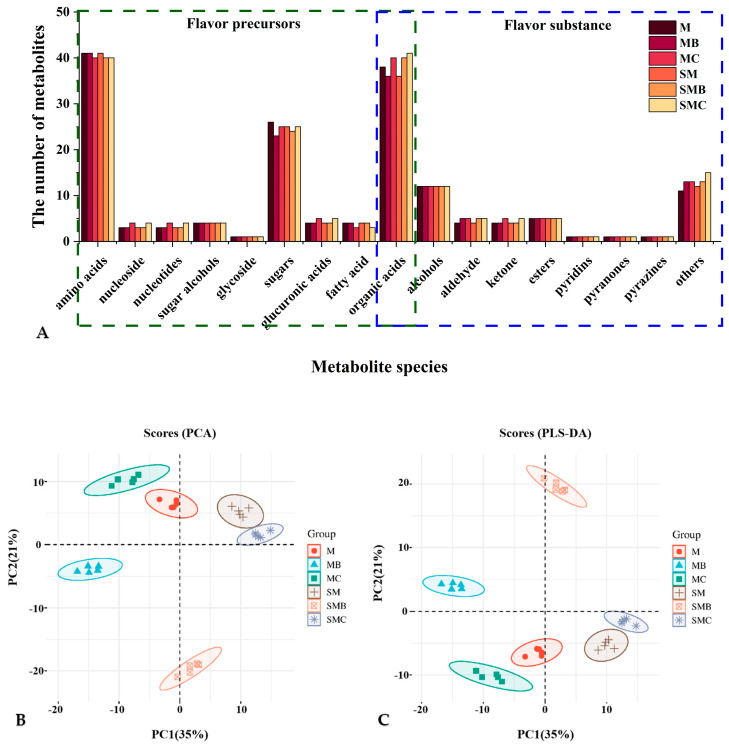
(**A**) Comparison of the metabolite numbers among fresh mung beans (M), baked mung beans (MB), boiled mung beans (MC), stored mung beans (SM), baked mung beans (SMB), and boiled mung beans (SMC). (**B**,**C**) PCA and PLS-DA score scatter plots.

Utilising a GC-MS-based untargeted metabolomics approach, 176 flavour-related metabolites were identified across the sample/groups M, MB, MC, SM, SMB, and SMC, accounting for 51.61% of the total metabolites. The identified metabolites encompassed 131 flavour precursor compounds, including amino acids and their derivatives, sugars and their derivatives, organic acids and their derivatives, fatty acids and their derivatives, and nucleosides and their derivatives. In addition, 45 flavour compounds, including alcohols, aldehydes, ketones, esters, pyridines, pyrazines, and lactones, were identified (Figure 1A). These results indicated the presence of three unique metabolites in fresh mung beans: thymol, pyrrole-2-carboxylic acid, and L-cysteine. These metabolites have been shown to play critical roles in sensory attributes, preservation, and antibacterial efficacy. Conversely, tyramine, a compound with the potential to compromise human health, was detected in stored mung beans. Following the processing of mung beans, seven precursor compounds and six characteristic flavour compounds emerged. Roasting resulted in the generation of flavour-enhancing compounds, such as gluconic acid 1 and pyrrole-2-carboxylic acid, whereas boiling helped to form a ketone compound, 3-hydroxy-2-butanone. Both processing methods helped to form key caramelisation compounds, such as 2-furoic acid and aroma compounds derived from the Maillard reaction, including E-2-hexenal. Compared to fresh mung beans, the generation of these volatile flavour compounds is believed to be associated with the anabolic metabolism of proteins, TAA, carbohydrates, fats, and their derivatives [19]. However, storage did not modify the types of flavour compounds.

### 3.2. Multivariate Analysis of Metabolites Associated with Flavour in Mung Beans

**Figure 2:**
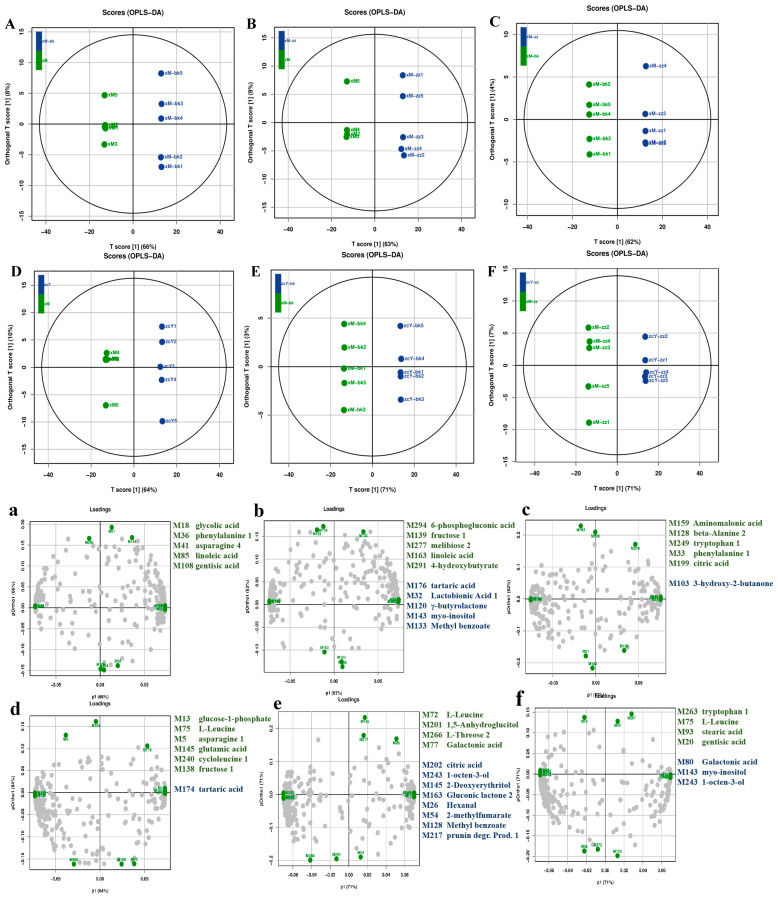
(**A**–**F**) OPLS-DA score scatter plots for xM(M), xM-bk(MB), xM-zz(MC), zcY(SM), zcY-bk(SMB), and zcY-zz(SMC). (**a**–**f**) OPLS-DA loading scores for xM(M), xM-bk(MB), xM-zz(MC), zcY(SM), zcY-bk(SMB), and zcY-zz(SMC).

PCA and partial least squares-discriminant analysis (PLS-DA) were used to analyse the flavour-related metabolic data across six sample groups to evaluate their contribution to the mung bean flavour value model. As shown in Figure 1B,C, the differences among the six groups were distinguished in the PCA and PLS-DA score plots. The PC1 and PC2 scores accounted for 35% and 21% of the total variance, respectively, with R2X = 0.558 > 0.5. Considering the variability among the samples, OPLS-DA was further applied to perform cross-validation of the differential metabolites in each sample group, providing more distinct classification results and higher predictive accuracy. As shown in Figure 2A–F, the R2X, R2Y, and Q2 values of the OPLS-DA models for each group were all greater than 0.5, with Q2 values exceeding 0.990(for the M vs. SM group, R2Y = 0.741, R2Y = 0.999, Q2 = 0.992; for the M vs. MB group, R2Y = 0.741, R2Y = 1, Q2 = 0.996; for the M vs. MC group, R2Y = 0.723, R2Y = 1, Q2 = 0.992; for the MB vs. MC group, R2Y = 0.664, R2Y = 1, Q2 = 0.994; for the MB vs. SMB group, R2Y = 0.742, R2Y = 1, Q2 = 0.997; for the MC vs. SMC group, R2Y = 0.786, R2Y = 1, Q2 = 0.997), and for all six sample groups, the R2Y and Q2 values were lower than the original R2Y and Q2 values obtained from permutation tests. This indicates that the models were free from overfitting and exhibited strong predictive accuracy [20]. Comprehensive analysis revealed good clustering within each sample group, demonstrating that the data processing was consistent and reliable. However, significant dispersion among the groups suggested that processing methods and storage had a noticeable effect on the flavour quality of mung beans.

The OPLS-DA loading plots (Figure 2a–f) further revealed the contribution of endogenous metabolites to the classification of the six sample groups. Specifically, changes induced by processing primarily depended on substances, such as amino acids, organic acids, fatty acids, and sugars, as well as volatile flavour compounds, including methyl benzoate, γ-butyrolactone, and 3-hydroxy-2-butanone. Changes caused by storage were strongly associated with amino acids, such as aspartic acid, glutamic acid, and leucine, which are mainly umami amino acids (Table 1), and branched-chain amino acids that can enhance aroma through biosynthesis. Furthermore, flavour compounds and precursor substances, such as hexanal, methyl benzoate, 1-octen-3-ol, prunin degradation product 1, gluconic lactone 2, 2-deoxyerythritol, and gentisic acid, significantly contributed to the differences between the SMB/SMC and MB/MC groups.

### 3.3. TAA Analysis of Six Groups of Mung Bean Samples Under Different Treatment Conditions

Based on the results of multivariate statistical analysis, the synthesis, degradation, and metabolism of TAA were likely one of the main reasons for the flavour differences observed in mung beans after processing and storage. The total amino acid content in the M, SM, MC, MB, SMC, and SMB samples was determined using an amino acid analyser, and their threshold aroma value was calculated (TAV > 1 indicates a contribution to flavour) [16]. This evaluation assessed their impact on the overall flavour characteristics of mung beans. As shown in Table 1, 44.53 ± 1.33% of TAA in fresh mung beans exhibited bitterness, with the highest TAV observed for arginine (Arg), indicating its significant contribution to the bitterness of mung beans. After roasting and boiling, the total amount of bitter amino acids decreased significantly, likely due to glycation, degradation, or evaporate of amino acids during processing. Amjad et al. found that phenylalanine, a bitter amino acid, underwent Strecker degradation during roasting to produce volatile aldehyde flavour compounds [21]. In addition, boiling reduced the content of water-soluble TAA, such as leucine (Leu) and arginine (Arg) [22]. A comparison of mung bean samples before and after storage revealed that the proportion of umami amino acids in the stored samples (SM) decreased significantly, whereas the proportion of bitter amino acids increased. This phenomenon may result from protein degradation into small peptides or TAA under the action of microproteases, as well as the consumption of some amino acids by microorganisms. Previous studies by our research group have also found that the protein content in mung beans initially decreases and then increases during storage [23]. Although the total sweet taste value (TST) of sweet amino acids increased significantly in processed mung beans after storage, it remained lower than the TST value of processed fresh mung beans. The total fresh taste value (TFT) of umami amino acids decreased significantly, whereas the total bitter taste value (TBT) of bitter amino acids increased significantly. These findings indicate that the use of stored mung beans for food preparation severely affects the flavour and nutritional characteristics.

### 3.4. Differential Metabolite Analysis

Further investigation of the effects of the six treatment groups on flavour precursors and differential flavour metabolites in mung beans revealed significant differences in the types and contents of flavour precursors and volatile flavour compounds under different cooking methods (Table 2). Specifically, 44 and 57 flavour precursor compounds were downregulated in roasted and boiled mung beans, respectively, with amino acids, sugars, and organic acids as primary differential metabolites. As reducing sugars and amino acids serve as precursors for the Maillard reaction, their levels decrease during thermal processing [24]. In addition, the loss of flavour precursors during boiling is primarily due to their dissolution in water under wet heat-processing conditions, leading to a more pronounced reduction. In the MB group, 22 volatile flavour compounds were upregulated, whereas 18 volatile flavour compounds were upregulated in the MC group. Baking resulted in a significant upregulation of 21 volatile flavour compounds compared with boiling, thus imparting roasted mung beans with a richer flavour profile. These included buttery aromas from aldehydes, fresh aromas from alcohols, and fruity notes from esters [25], which were primarily attributed to caramelisation and advanced Maillard reactions during baking [26]. In contrast, boiled mung beans are characterised by mild sweetness and umami notes [27]. When comparing mung beans stored for 1 year with fresh mung beans, the content of 13 amino acids decreased, whereas that of 10 amino acids increased, consistent with the trends observed in the free amino acid analysis. This can mainly be attributed to natural metabolic activity, respiration, and microbial activity during storage [28]. Under identical baking conditions, the volatile flavour compounds in the SMB group showed upregulation of eight compounds and downregulation of 16 compared to those in the MB group. The consumption or transformation of sugars during storage due to respiration and microbial metabolism leads to a reduction in the sugar substrates available for caramelisation reactions compared to fresh mung beans. Consequently, the intensity of caramel-like aromas was weaker in the stored mung beans. In the SMC group, compared to the MC group, sweet-flavour compounds, such as xylitol, sorbitol, mannitol, 1,3-cyclohexanedione 2, diglycerol 1, ribonic acid gamma-lactone, gluconic lactone 2, gluconic lactone 1, and D-erythronolactone 2, were upregulated. The SMC group exhibited higher sweetness than the MC group did. However, a decrease in compounds, such as 4-methylbenzyl alcohol, 1-hexadecanol, 1-octen-3-ol, 2,3-butanedione, and 2-methoxy-3-isobutyl-pyrazine, led to a reduction in flavour complexity (Supplementary Tables S1 and S2).

**Figure 3:**
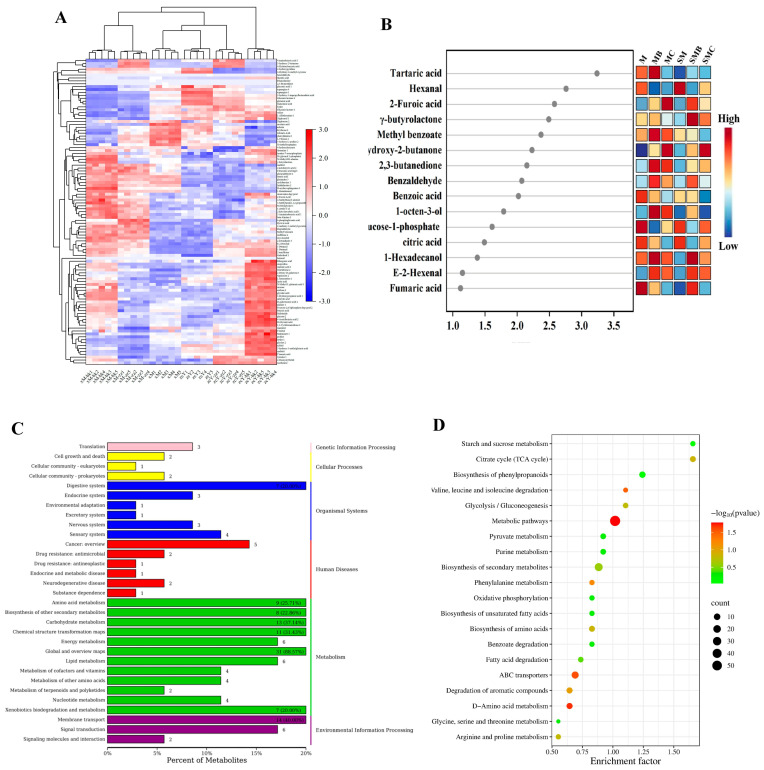
Analysis of M, MB, MC, SM, SMB, and SMC differential metabolites. (**A**) Hierarchical cluster analysis (HCA) and heat maps. (**B**) Important volatiles (VIP > 1.0) were identified using PLS-DA. (**C**) Significant enrichment analysis of KEGG and (**D**) metabolic pathways.

The heatmap (Figure 3A) revealed that after thermal processing, the content of most TAA, organic acids, and sugars in mung beans decreased, mainly because of the thermal decomposition of oligosaccharides in legumes to produce reducing sugars [29]. Reducing sugars and TAA undergo Maillard reactions to generate volatile compounds. After heat treatment, the levels of furans, pyrazines, ketones, aldehydes, alcohols, and esters in mung beans increased significantly, with the main significant differences being 2-amylfuran (liquorice flavour), 2-methoxy-3-isobutyl-pyrazine (green pepper, coffee flavour), 2,3-butanedione (buttery aroma), methyl benzoate (winter green and ylang-ylang oil aroma), E-2-hexenal (green flavour), and 1-octen-3-ol (strong, distinctive green aroma with a rice-bran-like odour) [30,31,32,33]. However, although 2-hydroxypyridine and 2-methoxy-3-isobutyl-pyrazine increased in both the baking and boiling groups, the increase was more significant in the MB group, mainly because the Maillard reaction occurred under baking conditions. The intermediate products of the Maillard reaction can further react with Strecker degradation and lipid oxidation products to form complex secondary products, such as pyridines and pyrazines. Therefore, the flavour of roasted mung beans is better than that of boiled mung beans.

In addition, as shown in Figure 3A, the contents of carbohydrates and amino acids (aspartic acid, leucine, glutamic acid, glycine, valine, and tyrosine) in mung beans decreased after storage, whereas those of sugar alcohols, uronic acids, and amines increased. This may be due to the partial degradation of starch into sugars under the action of amylase in mung beans and of free sugars into sugar derivatives during storage. Consequently, sugar and sugar derivatives increase after storage [34]. The decomposition of proteins and amino acids during storage can also affect the flavour of mung beans. From the figure, it can be observed that substances with specific aromatic properties, such as xylitol (fruit-like aroma) and methyl benzoate (wintergreen and ylang-ylang oil aroma), showed a decreasing trend after storage, whereas off-flavour compounds like hexanal exhibited an increasing trend. Consequently, the flavour complexity and palatability of mung beans decreased after storage. Heat processing of mung beans after storage also produced flavour compounds; however, compared with the heat processing of fresh mung beans, the contents of flavour compounds, such as aldehydes, ketones, pyridines, and pyrazines, significantly decreased, whereas amino acids and lactone compounds significantly increased.

The VIP values reflect the importance of each component in PLS-DA in a two-dimensional space. A VIP value greater than one indicates that the substance plays an important role in the corresponding sample; the larger the VIP value, the greater its importance [35]. Figure 3A shows the top 15 VIP values for each group, and it can be observed that the top three were tartaric acid, hexanal, and 2-furoic acid, which are consistent with the heatmap distribution results.

### 3.5. KEGG Annotation and Enrichment Analysis of Differential Metabolites

Metabolite pathway enrichment screening and identification were conducted using the KEGG pathway database, which revealed that all differential metabolites among the six sample groups were involved in 145 metabolic pathways. Among these, the most enriched pathways were related to metabolic pathways (M) and biosynthesis (O) (Figure 3C). Based on a comprehensive analysis of the-log(p) and enrichment factor values, the 20 most significant metabolic pathways were identified. The key metabolic pathways, as visualised in Figure 3D, D-amino acid metabolism, phenylalanine metabolism, the citrate cycle (TCA cycle), biosynthesis of phenylpropanoids, arginine and proline metabolism, glycolysis/gluconeogenesis, fatty acid degradation, benzoate degradation, and pyruvate metabolism, among others. By integrating the dominant metabolites identified through VIP values, such as benzoic acid, hexanal, γ-butyrolactone, methyl benzoate, tartaric acid, 1-octen-3-ol, glucose-1-phosphate, and citric acid (Figure 3B), it was found that the production of flavour compounds primarily depended on four metabolic pathways: glycolysis, citric acid, amino acid, and fatty acid degradation. In addition, seven other dominant metabolites identified in VIP analysis were closely associated with the Maillard reaction. Therefore, exploring the major pathways and chemical reactions mentioned above is critical to understand the mechanisms underlying the formation of flavour compounds.

**Figure 4:**
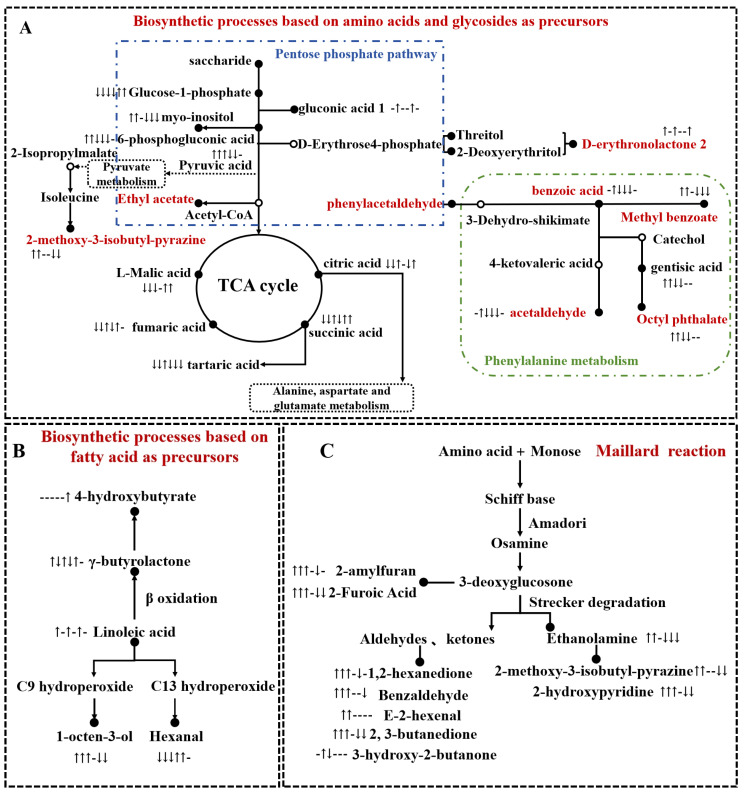
Analysis of the relationship between important flavour precursors and related flavour substances based on the KEGG pathway and Maillard reaction. The arrows in each differential metabolite in the Figure represent ↑ expression upregulation, ↓ expression downregulation, and - not significant, respectively. The order from left to right is M vs. MB, M vs. MC, MB vs. MC, M vs. SM, MB vs. SMB, and MC vs. SMC. Black circles represent the relevant differential metabolites in metabolic processes.

**Figure 5:**
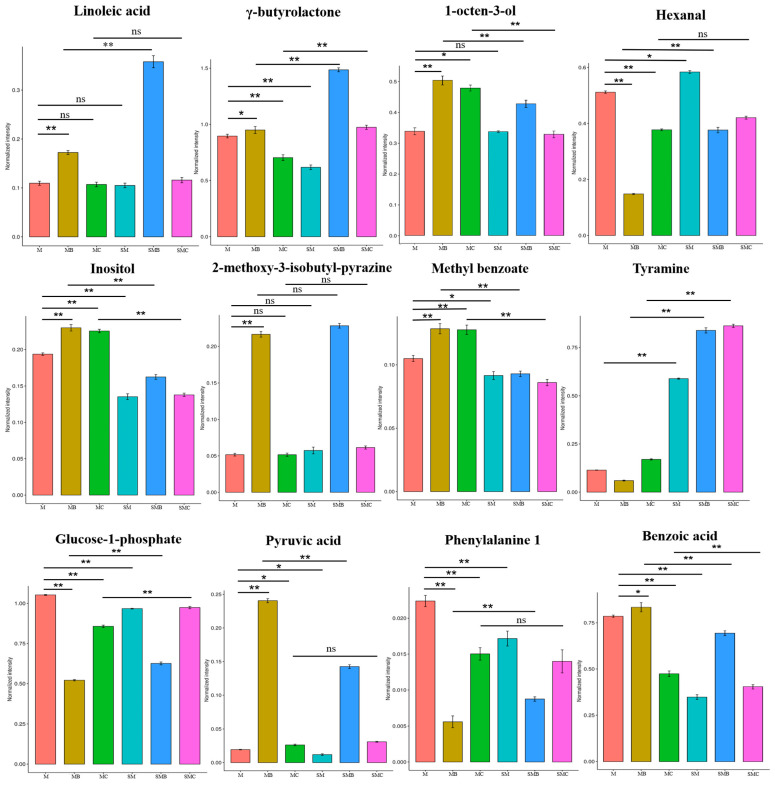
Normalisation intensity analysis of active volatile compounds as key characteristic flavours and their precursor metabolites in fresh, processed, stored, and processed mung beans. “*” indicates *p* < 0.05, “**” indicates *p* < 0.01, and “ns” indicates not significant.

As shown in Figure 4A, eight flavour compounds, ethyl acetate, phenylacetaldehyde, myo-inositol, 2-methoxy-3-isobutyl-pyrazine, D-erythronolactone 2, acetaldehyde, octyl phthalate, and methyl benzoate, were produced through the pentose phosphate pathway (pyruvate metabolism/amino acid metabolism) from sugar-based precursors. As a precursor for flavour formation, the content of glucose-1-phosphate in fresh mung beans significantly decreased after processing, whereas pyruvic acid content showed an increasing trend after processing (Figure 5). Baking has a significant impact, likely due to the high temperatures during baking, which favour the accumulation of pyruvic acid [36]. However, after storage, the consumption of glucose-1-phosphate and the accumulation of pyruvic acid during processing decreased significantly, indicating that storage substantially affected the metabolism and downstream transformation of flavour precursors. The myo-inositol content in stored mung beans decreased significantly, whereas that of 2-methoxy-3-isobutyl-pyrazine increased significantly (Figure 5). This may be because during storage, precursor compounds undergo continuous enzymatic degradation, favouring the production of 2-methoxy-3-isobutyl-pyrazine, leading to a reduction in myo-inositol content [37]. Studies have shown that 2-methoxy-3-isobutyl-pyrazine is associated with a grassy aroma [38]. After baking, the content of 2-methoxy-3-isobutyl-pyrazine in mung beans increased significantly, contributing to the overall enhancement of mung bean flavour quality and sugar metabolism. Phenylalanine metabolism also significantly influenced the flavour of mung beans. As shown in Figure 4A and Figure 5, storage negatively affected the retention of phenylalanine 1, whereas baking and boiling accelerated the degradation of phenylalanine 1. This promotes the accumulation of intermediate products, such as benzoic acid and downstream volatile flavour compounds, including methyl benzoate, octyl phthalate, and acetaldehyde [39]. Although the trends in benzoic acid content differed between baking and boiling, they may still be attributed to temperature variations during processing.

Fatty acids, which are highly oxidising substances, usually produce unpleasant odours, such as rancid and sour, during grain storage [40,41]. As shown in Figure 4B, γ-butyrolactone, 1-octen-3-ol, hexanal, and 4-hydroxybutyrate are flavour compounds synthesised and transformed from linoleic acid by lipoxygenase, cleavage enzymes, isomerase, oxidase, and other enzymes. Among these, hexanal is considered a key flavour compound responsible for the “beany flavour” [42]. After steaming and baking, the hexanal content of the mung beans decreased significantly. Fresh mung beans subjected to baking exhibited the lowest hexanal content, which may be influenced by the temperature during processing [43]. High temperatures during baking can cause the rapid dehydration of the mung bean surface, forming a hard crust that limits oxygen penetration and inhibiting the oxidation of linoleic acid to some extent. In contrast, during boiling, the temperature is lower and prolonged heating facilitates the gradual progression of linoleic acid oxidation, helping to generate more hexanal [40]. After storage, the hexanal content of mung beans increased significantly, whereas the change in the 1-octen-3-ol content was not significant. This may be because enzyme activity in mung beans increases during storage, accelerating the hydrolysis rate of C13 hydroperoxides, contributing to an increase in the hexanal content (Figure 5). Ren et al. [44] suggested that 1-octen-3-ol may cause unpleasant sensory perception. Therefore, the increased contents of hexanal and 1-octen-3-ol during storage may reduce consumer acceptance. The flavour characteristics of mung beans can only be improved through specific processing methods.

The Maillard reaction and thermal degradation are key pathways for the formation of flavour compounds [43]. In mung beans, starch is partially broken down into reducing sugars under the action of endogenous enzymes and heat treatment, which then reacts with TAA and peptides. As illustrated in Figure 4C, the amino acids in mung beans underwent nucleophilic addition reactions with monosaccharides. N-glucosamine loses a water molecule and undergoes Schiff base formation and Amadori rearrangement to produce 3-deoxyglucosone. Subsequent Strecker degradation reactions within the Maillard reaction produce a variety of flavour compounds, including aldehydes (Benzaldehyde, E-2-hexenal), ketones (1,2-hexanedione, 2,3-butanedione, and 3-hydroxy-2-butanone), enolamines (ethanolamine), furan derivatives (2-amylfuran, 2-Furoic Acid), and pyridine derivatives (2-hydroxypyridine). These molecular rearrangements, dehydration, and degradation processes in the Maillard reaction provide a foundation for the formation of volatile aromatic compounds [45]. The interplay between thermal treatment and the Maillard reaction significantly contributes to the unique and desirable flavour profiles of mung beans after processing. This highlights the importance of optimising processing conditions to enhance the diversification and intensity of flavour compounds in mung bean products.

The formation of off-flavour compounds, such as hexanal and 1-octen-3-ol, in mung beans relies primarily on the fatty acid metabolism pathway. These metabolic pathways can be regulated through appropriate processing methods, such as baking and boiling, reducing the synthesis and transformation of off-flavour compounds. During the initial phase of thermal processing, enzymatic reactions are significantly enhanced, resulting in the release of sugars and amino acids, and the concurrent initiation of the Maillard reaction. As the processing temperature increases, the mung beans are inactivated, yet the endogenous enzymes within the seeds remain partially active, leading to the release of additional sugars and amino acids. This further intensifies the Maillard reaction. In the middle and later stages of thermal treatment, the high temperature completely inactivates the endogenous enzymes, thereby terminating the release of flavour precursor compounds. Subsequently, the ongoing Maillard reaction begins to consume these flavour precursors. These methods can accelerate the consumption of sugar and amino acid precursors, promoting the formation of aromatic volatile compounds (E-2-hexenal, methyl benzoate, 2-methoxy-3-isobutyl-pyrazine, octyl phthalate, 2,3-butanedione, and others) via sugar metabolism, amino acid metabolism, and the Maillard reaction. However, the storage process significantly impacts the content of precursor compounds, such as amino acids, fatty acids, and sugars in mung beans. This can cause insufficient synthesis of key flavour compounds during subsequent processing, affecting the overall flavour profile of mung beans. In addition, lipid oxidation during storage can accelerate the formation of off-flavour compounds and produce potentially harmful substances such as biogenic amines (tyramine), which may pose risks to human health. To ensure high-quality flavour and safety in mung bean products, it is essential to optimise storage conditions (controlling temperature, humidity, and oxygen exposure) and apply effective processing methods to balance the reduction in off-flavours and enhance desirable aroma compounds. This integrated approach can improve consumer acceptance, while maintaining the nutritional and sensory qualities of mung bean products.

## 4. Conclusions

This study analysed the dynamic changes in aroma compounds, precursors, and major metabolic pathways in six sample groups (M, MB, MC, SM, SMB, and SMC) and investigated the main mechanisms of flavour formation. A total of 176 flavour-related metabolites were detected using GC-MS. Storage reduced the umami amino acid content and increased the bitter amino acid content. The impact of thermal processing on flavour generation was also examined. Baking tends to generate higher levels of aldehyde, alcohol, and ester compounds, which are associated with buttery, fresh, and fruity aromas. In contrast, boiling primarily promotes the formation of mild sweet and umami compounds. Under identical thermal treatment conditions, the SMB group exhibited lower levels of compounds potentially associated with caramel aroma compared to the MC group, whereas the SMC group had higher levels of these compounds. The analysis revealed that differential metabolites were implicated in 145 metabolic pathways, with flavour compounds predominantly produced through glycolysis, citric acid metabolism, amino acid metabolism, and fatty acid degradation. The analysis revealed the generation of ethyl acetate, phenylacetaldehyde, myo-inositol, 2-methoxy-3-isobutyl-pyrazine, D-erythronolactone 2, acetaldehyde, octyl phthalate, and methyl benzoate from carbohydrates via the pentose phosphate, pyruvate, and amino acid metabolism pathways. The formation of the mung beans’ off-flavour compounds hexanal and 1-octen-3-ol primarily relies on the fatty acid metabolic pathway, which provides theoretical guidance for the formation of mung bean flavour and scientific cooking and offers a scientific basis for elucidating the flavour mechanism and constructing a quality system for mung bean flavour.

## Figures and Tables

**Table 1:** Total amino acids content in M, MB, MC, SM, SMB, and SMC.

Flavour	Free Amino Acid	Content (g/100 g)					
		M	MB	MC	SM	SMB	SMC
Sweetness (S)	Ser	0.17 ± 0.01 ^c^	0.29 ± 0.04 ^a^	0.24 ± 0.02 ^b^	0.15 ± 0.01 ^d^	0.23 ± 0.01 ^b^	0.25 ± 0.02 ^ab^
	Gly	4.26 ± 0.12 ^d^	5.16 ± 0.05 ^a^	5.09 ± 0.03 ^b^	4.15 ± 0.07 ^e^	4.53 ± 0.15 ^d^	4.77 ± 0.04 ^c^
	Ala	8.10 ± 0.03 ^bc^	8.17 ± 0.01 ^a^	8.03 ± 0.04 ^d^	8.15 ± 0.06 ^ab^	8.10 ± 0.03 ^bc^	8.07 ± 0.09 ^bcd^
	Thr	1.08 ± 0.15 ^b^	1.10 ± 0.03 ^b^	1.45 ± 0.04 ^a^	0.99 ± 0.09 ^b^	1.01 ± 0.05 ^b^	1.12 ± 0.02 ^b^
Freshness (F)	Glu	7.54 ± 0.04 ^a^	7.39 ± 0.06 ^b^	7.24 ± 0.12 ^b^	7.04 ± 0.04 ^c^	5.87 ± 0.14 ^e^	6.59 ± 0.05 ^d^
	Asp	3.84 ± 0.17 ^cd^	4.72 ± 0.10 ^a^	4.04 ± 0.05 ^b^	3.77 ± 0.07 ^d^	3.99 ± 0.06 ^bc^	3.87 ± 0.11 ^cd^
Bitterness (B)	Val	4.67 ± 0.11 ^ab^	4.71 ± 0.06 ^a^	4.63 ± 0.17 ^b^	4.61 ± 0.12 ^bc^	4.53 ± 0.02 ^c^	4.59 ± 0.05 ^b^
	Met	1.08 ± 0.08 ^b^	0.84 ± 0.02 ^c^	0.73 ± 0.01 ^d^	2.05 ± 0.07 ^a^	2.12 ± 0.04 ^a^	2.10 ± 0.02 ^a^
	Ile	4.28 ± 0.07 ^a^	2.97 ± 0.12 ^f^	3.75 ± 0.04 ^d^	4.12 ± 0.04 ^b^	3.24 ± 0.03 ^e^	4.01 ± 0.09 ^c^
	Leu	9.23 ± 0.05 ^a^	8.77 ± 0.02 ^c^	8.43 ± 0.11 ^d^	9.27 ± 0.10 ^a^	9.02 ± 0.05 ^b^	8.79 ± 0.03 ^c^
	Phe	3.76 ± 0.02 ^b^	2.88 ± 0.04 ^e^	2.54 ± 0.07 ^f^	3.92 ± 0.08 ^a^	3.03 ± 0.03 ^d^	3.25 ± 0.11 ^c^
	His	1.94 ± 0.02 ^b^	1.92 ± 0.04 ^bc^	2.01 ± 0.06 ^a^	1.96 ± 0.01 ^ab^	1.91 ± 0.01 ^c^	1.89 ± 0.01 ^d^
	Arg	6.85 ± 0.03 ^a^	5.76 ± 0.07 ^e^	5.74 ± 0.03 ^e^	6.45 ± 0.05 ^c^	6.22 ± 0.01 ^d^	6.56 ± 0.06 ^b^
Tasteless and medicinal (T)	Pro	3.19 ± 0.07 ^b^	3.42 ± 0.04 ^a^	3.23 ± 0.07 ^b^	3.09 ± 0.04 ^cd^	3.05 ± 0.02 ^d^	2.97 ± 0.04 ^e^
	Tyr	4.57 ± 0.05 ^c^	4.61 ± 0.08 ^c^	4.84 ± 0.1 ^a^	4.42 ± 0.08 ^d^	4.60 ± 0.04 ^c^	4.75 ± 0.03 ^b^
	Lys	6.87 ± 0.04 ^c^	7.07 ± 0.02 ^a^	6.99 ± 0.09 ^ab^	6.82 ± 0.06 ^cd^	6.77 ± 0.14 ^d^	6.92 ± 0.09 ^b^
S/T (%)		19.05 ± 0.27 ^c^	21.09 ± 0.11 ^a^	21.47 ± 0.42 ^a^	18.94 ± 0.77 ^c^	20.33 ± 0.34 ^b^	20.16 ± 0.68 ^b^
F/T (%)		15.93 ± 0.24 ^b^	17.35 ± 0.48 ^a^	16.35 ± 0.67 ^ab^	15.23 ± 0.44 ^c^	14.45 ± 0.38 ^d^	14.84 ± 0.43 ^cd^
B/T (%)		44.53 ± 1.33 ^ab^	39.91 ± 1.69 ^c^	40.35 ± 1.04 ^c^	45.63 ± 1.98 ^a^	44.08 ± 1.83 ^b^	44.24 ± 1.32 ^ab^
T/T (%)		20.48 ± 0.65 ^c^	21.64 ± 0.37 ^ab^	21.83 ± 0.13 ^a^	20.19 ± 0.57 ^b^	21.14 ± 0.41 ^b^	20.77 ± 0.72 ^c^
TST		179.01 ± 1.21 ^e^	189.24 ± 1.33 ^a^	187.28 ± 0.98 ^b^	178.37 ± 1.03 ^e^	181.60 ± 1.42 ^d^	183.84 ± 0.78 ^c^
TFT		289.73 ± 0.99 ^b^	293.53 ± 1.26 ^a^	281.73 ± 1.53 ^c^	272.37 ± 1.36 ^d^	235.57 ± 1.96 ^f^	258.37 ± 1.31 ^e^
TBT		946.05 ± 4.71 ^a^	806.68 ± 3.98 ^d^	810.48 ± 4.26 ^d^	938.37 ± 5.03 ^b^	898.30 ± 4.00 ^c^	940.45 ± 2.03 ^ab^

Note: Total flavoured amino acid TAV value, TFT; total bitter amino acid TAV value, TBT; total sweetened amino acid TAV value, TST; taste thresholds for Glu, Asp, Arg, His, Met, Ile, Leu, Phe, Trp, Val, Ala, Gly, Ser, and Thr were 30, 100, 10, 20, 30, 90, 380, 150, 90, 150, 60, 110, 150, and 260 (mg/100 g). Different letters (a–f) indicate significant differences between the mean values of different samples (*p* < 0.05).

**Table 2:** Statistical results for differential metabolite quantities.

Substances	MB vs. M			MC vs. M			MB vs. MC			SM vs. M			SMB vs. MB			SMC vs. MC		
	↓	↑	-	↓	↑	-	↓	↑	-	↓	↑	-	↓	↑	-	↓	↑	-
Amino acid	20	14	8	22	6	14	12	9	21	13	10	19	10	20	12	12	16	14
Nucleoside	0	2	0	1	0	1	0	0	2	1	1	0	1	1	0	1	1	0
Nucleotides	0	3	0	1	2	0	2	1	0	1	1	1	1	0	2	1	0	2
Sugar alcohols	0	3	0	0	1	2	0	2	1	1	1	1	1	0	2	1	2	0
Glycoside	1	0	0	1	0	0	1	0	0	0	0	1	0	0	1	0	0	1
Sugars	9	8	7	11	5	8	8	9	7	7	9	8	5	8	11	6	9	9
Glucuronic acids	3	1	1	4	0	1	1	4	0	1	2	2	0	5	0	0	3	2
Fatty acid	0	1	3	1	0	3	0	2	2	0	2	2	2	1	1	2	1	1
Organic acids	11	17	9	16	11	10	10	21	6	12	10	15	13	10	14	8	16	13
Alcohols	1	5	4	3	3	4	0	8	2	3	3	4	5	3	2	4	3	3
Aldehyde	1	5	0	1	5	0	1	2	3	0	2	4	2	1	3	3	0	3
Ketone	0	4	1	0	5	0	1	4	0	0	1	4	4	0	1	1	1	3
Esters	2	3	0	2	2	1	1	3	1	3	1	1	1	1	3	2	1	2
Pyridins	0	1	0	0	1	0	0	1	0	0	0	1	1	0	0	1	0	0
Pyranones	0	0	1	0	0	1	0	0	1	0	1	0	1	0	0	1	0	0
Pyrazines	0	1	0	0	1	0	0	0	1	0	0	1	1	0	0	1	0	0
Lactones	3	2	0	4	0	1	2	2	1	2	1	2	0	3	2	0	4	1
Furan derivatives	0	1	0	0	1	0	0	1	0	0	0	1	1	0	0	0	0	1
Total volatile flavour substance	7	22	6	10	18	7	5	21	9	8	9	18	16	8	11	13	9	13

Note: The “↑” represents an increase in the number of metabolites, the “↓” represents a decrease in the number of metabolites, and “-” means the number of metabolites was constant.

## Data Availability

The original contributions presented in the study are included in the article/Supplementary Material, further inquiries can be directed to the corresponding author.

## Supplementary Materials

The following supporting information can be downloaded at: https://www.mdpi.com/article/10.3390/foods14050797/s1, Table S1: Flavor difference metabolites for fresh mung bean after baking and cooking processing; Table S2: Flavor difference metabolites for mung beans and their processed products before and after storage.

## Abbreviations

The following abbreviations are used in this manuscript:

EDM	Euclidean Distance Matrix
TAA	Total amino acids
HCA	Hierarchical Cluster Analysis
KEGG	Kyoto Encyclopedia of Genes and Genomes
PCA	Principal component analysis
TBT	Total Bitter Taste
TFT	Total Fresh Taste
TST	Total Sweet Taste
VIP	Variable Importance in Projection
