# Supplementary material for: Metabolomic Analysis of Flavour Development in Mung Bean Foods: Impact of Thermal Processing and Storage on Precursor and Volatile Compounds
Source: Foods. 2025 Feb 26;14(5):797. doi: 10.3390/foods14050797 (PMC11899303; doi:10.3390/foods14050797)
Supplement: Supplementary file 1 [file foods-14-00797-s001.zip › foods-3443838-supplementary.pdf]

## Table S1 Flavor difference metabolites for fresh mung bean after baking and cooking processing

| Type | Peak | xM-bk vs. xM | | xM-zz vs. xM | | xM-bk vs. xM-zz | |
|---|---|---|---|---|---|---|---|
| | | FoldChange | significant | FoldChange | significant | FoldChange | significant |
| Amino acid | asparagine 1 | 2.72 | Down | 1.97 | Down | 0.73 | Down |
| | L-homoserine 1 | 0.72 | Up | - | - | 1.48 | Up |
| | aspartic acid 1 | 1.14 | Down | 1.45 | Down | 1.27 | Up |
| | glycine 2 | 1.49 | Down | 1.52 | Down | - | - |
| | serine 1 | 1.53 | Down | 1.46 | Down | - | - |
| | phenylalanine 1 | 2.02 | Down | 1.23 | Down | 0.61 | Down |
| | asparagine 4 | 3.45 | Down | 1.36 | Down | 0.39 | Down |
| | N-Methyl-DL-alanine | 0.57 | Up | 1.22 | Down | 2.12 | Up |
| | citrulline 1 | 1.99 | Down | 1.46 | Down | 0.73 | Down |
| | O-Phosphoserine 1 | 1.44 | Down | - | - | - | - |
| | glycine 1 | 0.78 | Up | 1.46 | Down | 1.88 | Up |
| | canavanine degr prod | 0.70 | Up | 0.80 | Up | - | - |
| | 4-aminobutyric acid 1 | 1.91 | Down | 0.67 | Up | 0.35 | Down |
| | Norleucine 2 | 0.72 | Up | - | - | - | - |
| | valine | 1.33 | Down | 1.27 | Down | - | - |
| | glycocyamine 3 | 0.39 | Up | - | - | 1.58 | Up |
| | O-acetylserine 1 | 0.73 | Up | - | - | - | - |
| | beta-Alanine 2 | 0.74 | Up | 0.80 | Up | - | - |
| | N-methyltryptophan | 4.40 | Down | 1.79 | Down | 0.34 | Down |
| | glutamic acid | 2.12 | Down | 1.23 | Down | 0.58 | Down |
| | Aminomalonic acid | 1.38 | Down | 1.38 | Down | - | - |
| | 3-Cyanoalanine | 5.32 | Down | 1.29 | Down | 0.24 | Down |
| | oxoproline | 0.31 | Up | - | - | 3.33 | Up |
| | N-Methyl-L-glutamic acid 3 | 0.53 | Up | - | - | 1.78 | Up |
| | lysine | 1.84 | Down | 1.71 | Down | - | - |
| | proline | 1.20 | Down | 1.61 | Down | 1.34 | Up |
| | 3-hydroxy-L-proline 2 | 2.20 | Down | 1.23 | Down | 0.56 | Down |
| | cycloleucine 1 | 0.92 | Up | 1.27 | Down | 1.37 | Up |
| | Isoleucine | 1.42 | Down | 1.21 | Down | 0.85 | Down |
| | N-Ethylglycine 1 | 0.67 | Up | 0.70 | Up | - | - |
| | 3-Indolepyruvic acid | 0.66 | Up | - | - | - | - |
| | tryptophan 1 | 2.68 | Down | 1.32 | Down | 0.49 | Down |
| | L-Allothreonine 1 | 1.56 | Down | 1.33 | Down | 0.85 | Down |
| | 3-Aminoisobutyric acid 1 | 0.69 | Up | 0.75 | Up | - | - |
| | N-Acetyl-5-hydroxytryptamine 1 | - | - | 0.80 | Up | - | - |
| | tyrosine 1 | - | - | 1.49 | Down | - | - |
| | 2-Amino-2-norbornanecarboxylic acid 1 | - | - | - | - | - | - |
| | glutamine 3 | - | - | - | - | - | - |

| | | | | | | | |
|---|---|---|---|---|---|---|---|
| | alanine 1 | - | - | - | - | - | - |
| | Maleamate 1 | - | - | - | - | - | - |
| | N-alpha-Acetyl-L-ornithine 1 | - | - | - | - | - | - |
| | threonine 1 | - | - | - | - | - | - |
| Nucleoside | xanthosine | 0.64 | Up | - | - | 1.29 | - |
| | Purine riboside | 0.64 | Up | 1.43 | Down | 2.25 | - |
| Nucleotides | 6-phosphogluconic acid | 0.14 | Up | 0.69 | Up | 5.02 | Down |
| | cytidine-monophosphate degr prod | 0.52 | Up | 0.58 | Up | - | Down |
| | inosine 5'-monophosphate | 0.90 | Up | 1.37 | Down | 1.52 | Up |
| Sugar alcohols | Galactinol 1 | 0.80 | Up | - | - | 1.18 | Up |
| | myo-inositol | 2.21 | Up | 2.28 | Up | - | - |
| | 2-Deoxyerythritol | 1.57 | Up | - | - | 1.80 | Up |
| Glycoside | arbutin | 2.05 | Down | 1.37 | Down | 0.67 | Down |
| Sugars | Glucose-1-phosphate | 1.14 | Down | 1.23 | Down | 1.08 | Down |
| | Sophorose 2 | 0.78 | Up | 0.85 | Up | 1.09 | Up |
| | D-Talose 2 | 2.24 | Down | 1.68 | Down | 0.75 | Down |
| | glucose 2 | 0.88 | Up | - | - | - | - |
| | sorbose 2 | 0.46 | Up | - | - | - | - |
| | fructose 1 | 1.30 | Down | - | - | 0.78 | Down |
| | Gentiobiose 2 | 0.73 | Up | 0.78 | Up | 1.07 | Up |
| | 2-Deoxy-D-galactose 1 | 0.55 | Up | 0.70 | Up | 1.26 | Up |
| | 2-deoxy-D-glucose 2 | 1.72 | Down | 1.34 | Down | 0.78 | Down |
| | Fructose 2,6-biphosphate degr prod 2 | 0.84 | Up | 1.18 | Down | 1.40 | Up |
| | 1,5-Anhydroglucitol | 1.34 | Down | 2.01 | Down | 1.49 | Up |
| | Erythrose 2 | 50.99 | Down | 2.30 | Down | 0.05 | Down |
| | mannose 1 | 4.33 | Down | 3.58 | Down | 0.83 | Down |
| | beta-Mannosylglycerate 2 | 1.43 | Down | 1.72 | Down | - | - |
| | L-Threose 2 | 1.95 | Down | 1.28 | Down | 0.66 | Down |
| | melibiose 2 | 0.61 | Up | 0.66 | Up | 1.09 | Up |
| | raffinose | 0.84 | Up | 1.40 | Down | 1.67 | Up |
| | D-Talose 1 | - | - | - | - | 0.79 | Down |
| | Lyxose 2 | - | - | 1.47 | Down | 1.32 | Up |
| | cellobiose 2 | - | - | - | - | 1.87 | Up |
| | Digitoxose 2 | - | - | - | - | - | - |
| | Threitol | - | - | - | - | - | - |
| | sucrose | - | - | - | - | - | - |
| | maltitol | - | - | 0.87 | Up | - | - |
| Glucuronic acids | Galactonic acid | 2.99 | Down | 1.80 | Down | 0.60 | Down |
| | D-galacturonic acid 2 | 0.74 | Up | 1.88 | Down | 2.17 | Up |
| | mucic acid | 2.07 | Down | 4.12 | Down | 1.99 | Up |
| | Saccharic acid | 1.72 | Down | 3.90 | Down | 2.27 | Up |

| | | | | | | | |
|---|---|---|---|---|---|---|---|
| | gluconic acid 1 | - | - | - | - | 0.50 | Up |
| | isocitric acid 2 | 0.90 | Up | 1.61 | Down | 1.78 | Up |
| | 3-Hydroxypropionic acid 1 | 0.24 | Up | - | - | 4.20 | Up |
| | Lactobionic Acid 1 | 0.82 | Up | 0.74 | Up | 0.90 | Down |
| | Itaconic acid | 1.64 | Down | - | - | 0.61 | Down |
| | lactic acid | 0.46 | Up | 0.70 | Up | 1.50 | Up |
| | Pyruvic acid | 0.08 | Up | 0.73 | Up | 9.17 | Up |
| | 2-ketoadipate 3 | 0.64 | Up | 0.73 | Up | 1.15 | Up |
| | maleic acid | 1.45 | Down | 1.72 | Down | 1.18 | Up |
| | succinic acid | 1.24 | Down | 1.65 | Down | 1.33 | Up |
| | fumaric acid | 1.52 | Down | 1.82 | Down | 1.20 | Up |
| | 2-ketobutyric acid 2 | 0.82 | Up | 0.82 | Up | - | - |
| | glycolic acid | 0.18 | Up | - | - | 5.50 | Up |
| | salicylic acid | 0.45 | Up | - | - | 2.49 | Up |
| | oxalic acid | 0.68 | Up | - | - | 1.29 | Up |
| | 2-keto-isovaleric acid 1 | 0.77 | Up | 0.84 | Up | - | - |
| | D-Glyceric acid | 0.83 | Up | 1.31 | Down | 1.57 | Up |
| | tartaric acid | 1.28 | Down | 2.33 | Down | 1.83 | Up |
| | malonic acid 1 | 0.51 | Up | - | - | 1.70 | Up |
| Organic acids | Indolelactate 2 | 0.80 | Up | - | - | 1.25 | Up |
| | 2-hydroxy-3-isopropylbutanedioic acid | 2.38 | Down | 2.08 | Down | 0.87 | Down |
| | citric acid | 1.54 | Down | 2.04 | Down | 1.28 | Up |
| | Citraconic acid degr1 | 0.80 | Up | 0.80 | Up | - | - |
| | Aconitic Acid | 1.85 | Down | 1.62 | Down | 0.87 | Down |
| | Glutaric Acid | 4.00 | Down | 1.49 | Down | 0.37 | Down |
| | L-Malic acid | 1.18 | Down | 2.54 | Down | 2.16 | Down |
| | gentisic acid | 0.15 | Up | 2.13 | Up | 1.44 | Down |
| | benzoic acid | - | - | 1.66 | Up | 1.76 | Down |
| | Threonic acid | - | - | 1.26 | Down | 1.25 | Up |
| | Tartronic acid | - | - | - | - | 0.72 | Down |
| | 3-hydroxy-3-methylglutaric acid | - | - | 1.49 | Down | 1.73 | Up |
| | Citramalic acid | - | - | 1.68 | Down | 1.49 | Up |
| | Dehydroascorbic Acid 1 | - | - | 1.65 | Down | - | - |
| | Pelargonic acid | - | - | - | - | - | - |
| | 4-Acetylbutyric acid 2 | - | - | - | - | - | - |
| | 4-Hydroxybenzoic acid | - | - | 0.69 | Up | 1.50 | Down |
| | 2-methylfumarate | 0.81 | Down | 1.16 | Down | 1.71 | Up |
| | 2-Furoic Acid | 1.60 | Up | 0.60 | Up | 2.16 | Up |
| | palmitic acid | - | - | 1.61 | Down | 1.66 | Up |
| Fatty acid | stearic acid | - | - | - | - | - | - |
| | linoleic acid | 0.64 | Up | - | - | 1.42 | Up |

| | | | | | | | |
|---|---|---|---|---|---|---|---|
| Alcohols | 4-hydroxybutyrate | - | - | - | - | - | - |
| | 4-Methylbenzyl alcohol | 0.68 | Up | 0.79 | Up | 1.16 | Up |
| | 1-Hexadecanol | 0.77 | Up | - | - | 1.25 | Up |
| | xylitol | 1.11 | Down | 1.37 | Down | 1.24 | Up |
| | D-erythro-sphingosine 2 | 0.58 | Up | - | - | 1.70 | Up |
| | sorbitol | - | - | 1.79 | Down | 1.73 | Up |
| | mannitol | - | - | 2.06 | Down | 1.94 | Up |
| | ribitol | - | - | - | - | 1.69 | Up |
| | Octadecanol | - | - | - | - | - | - |
| | 1-octene-3-ol | 1.24 | Up | 0.27 | Up | 0.96 | Up |
| | 1-amyl alcohol | 1.25 | Up | 1.48 | Up | - | - |
| Aldehyde | glutaraldehyde 4 | 0.84 | Up | 0.86 | Up | - | - |
| | benzaldehyde | 0.24 | Up | 0.33 | Up | 0.34 | Up |
| | Hexanal | 0.25 | Down | 0.47 | Down | 1.05 | Down |
| | enanthal | 1.44 | Up | 0.50 | Up | - | - |
| | 1-amyl alcohol | 0.46 | Up | 0.87 | Up | 2.08 | Up |
| | E-2-hexenal | 0.68 | Up | 0.64 | Up | - | - |
| Ketone | prunin degr. Prod. 1 | 0.61 | Up | 0.38 | Up | 0.67 | Up |
| | 1,2-Cyclohexanedione 4 | 0.75 | Up | 1.69 | Up | 1.48 | Up |
| | 1,3-Cyclohexanedione 2 | 0.91 | Up | 1.27 | Up | 1.35 | Up |
| | 2,3-Butanedione | 0.18 | Up | 0.73 | Up | 0.99 | Up |
| | 3-hydroxy-2-butanone | - | - | | Up | | Down |
| Esters | Dioctyl phthalate | 0.75 | Up | 0.91 | Up | 1.21 | Down |
| | Methyl Phosphate | 5.08 | Down | 6.48 | Down | 2.05 | Up |
| | Diglycerol 1 | 1.17 | Down | 1.52 | Down | 1.30 | Up |
| | D-(glycerol 1-phosphate) | 0.84 | Up | - | - | 1.16 | Up |
| | Methyl benzoate | 1.26 | Up | 1.63 | Up | - | - |
| Pyridins | 2-hydroxypyridine | 0.76 | Up | 0.88 | Up | 1.15 | Up |
| Pyranones | 2-methoxy-3-isobutyl-pyrazine | 0.78 | Up | 0.85 | Up | - | - |
| Pyrazines | 4-Hydroxy-6-methyl-2-pyrone | - | - | - | - | - | - |
| Lactones | Ribonic acid, gamma-lactone | 1.69 | Down | 1.44 | Down | 0.85 | Down |
| | Gluconic lactone 2 | 2.22 | Down | 1.73 | Down | 0.78 | Down |
| | Gluconic lactone 1 | 1.35 | Down | 1.54 | Down | - | - |
| | D-erythronolactone 2 | 1.47 | Up | - | - | 1.48 | Up |
| | γ-butyrolactone | 1.57 | Up | 2.57 | Down | 1.47 | Up |
| Furan derivatives | 2-amylfuran | 1.28 | Up | 0.98 | Up | 0.49 | Up |
| Amines | Ethanolamine | 1.87 | Down | 1.46 | Down | 0.78 | Down |
| | Tyramine | - | - | - | - | - | - |

**Table S2 Flavor difference metabolites for mung beans and their processed products before and after storage.**

| Type | Peak | zcY vs.xM | | zcY-bk vs. xM-bk | | zcY-zz vs. xM-zz | |
|---|---|---|---|---|---|---|---|
| | | FoldChange | significant | FoldChange | significant | FoldChange | significant |
| | asparagine 1 | 2.37 | Up | 0.43 | Up | 0.35 | Up |
| | L-homoserine 1 | 1.39 | Up | 0.73 | Up | - | - |
| | aspartic acid 1 | 0.85 | Down | 0.89 | Up | 0.94 | Up |
| | glycine 2 | 0.84 | Down | 0.35 | Up | 0.51 | Up |
| | serine 1 | - | - | 0.40 | Up | 0.59 | Up |
| | phenylalanine 1 | 0.92 | Down | 0.83 | Up | - | - |
| | asparagine 4 | 1.14 | Up | 0.39 | Up | 0.90 | Up |
| | N-Methyl-DL-alanine | 0.56 | Down | 1.43 | Down | 1.25 | Down |
| | citrulline 1 | 0.73 | Down | - | - | - | - |
| | O-Phosphoserine 1 | - | - | - | - | 2.68 | Down |
| | glycine 1 | 2.73 | Up | 1.20 | Down | 0.51 | Up |
| | canavanine degr prod | - | - | - | - | 1.32 | Down |
| | 4-aminobutyric acid 1 | 5.16 | Up | 0.07 | Up | 0.20 | Up |
| | Norleucine 2 | - | - | - | - | 1.35 | Down |
| | valine | - | - | 0.71 | Up | 0.81 | Up |
| | glycocyamine 3 | 1.53 | Up | 3.91 | Down | - | - |
| | O-acetylserine 1 | - | - | - | - | - | - |
| | beta-Alanine 2 | - | - | 1.22 | Down | 1.40 | Down |
| | N-methyltryptophan | 0.67 | Down | - | - | 0.82 | Up |
| Amino acid | glutamic acid | 2.29 | Up | 0.35 | Up | 0.44 | Up |
| | Aminomalonic acid | - | - | 0.77 | Up | - | - |
| | 3-Cyanoalanine | 0.60 | Down | 0.28 | Up | - | - |
| | oxoproline | 1.58 | Up | 0.66 | Up | 0.49 | Up |
| | N-Methyl-L-glutamic acid 3 | - | - | - | - | - | - |
| | lysine | - | - | 0.53 | Up | 0.48 | Up |
| | proline | - | - | 0.59 | Up | 0.56 | Up |
| | 3-hydroxy-L-proline 2 | 0.77 | Down | - | - | - | - |
| | cycloleucine 1 | 0.29 | Down | 2.07 | Down | 2.33 | Down |
| | Isoleucine | 0.74 | Down | 0.68 | Up | 0.80 | Up |
| | N-Ethylglycine 1 | - | - | - | - | 1.46 | Down |
| | 3-Indolepyruvic acid | - | - | 1.57 | Down | - | - |
| | tryptophan 1 | 0.30 | Down | 1.32 | Down | 2.13 | Down |
| | L-Allothreonine 1 | - | - | 0.51 | Up | 0.66 | Up |
| | 3-Aminoisobutyric acid 1 | 1.12 | Up | 1.21 | Down | 1.42 | Down |
| | N-Acetyl-5-hydroxytryptamine 1 | - | - | - | - | 1.69 | Down |
| | tyrosine 1 | 1.46 | Up | - | - | 0.36 | Up |
| | 2-Amino-2-norbornanecarb | - | - | 0.44 | Up | - | - |

| | | | | | | | |
|---|---|---|---|---|---|---|---|
| | oxylic acid 1 | | | | | | |
| | glutamine 3 | 0.58 | Down | 1.39 | Down | 2.07 | Down |
| | alanine 1 | - | - | 0.84 | Up | - | - |
| | Maleamate 1 | - | - | 0.63 | Up | - | - |
| | N-alpha-Acetyl-L-ornithine 1 | 0.72 | Down | 2.23 | Down | - | - |
| | threonine 1 | - | - | - | - | 1.38 | Down |
| Nucleoside | xanthosine | 1.27 | Down | 0.57 | Up | 0.75 | Up |
| | Purine riboside | 0.56 | Up | 2.15 | Down | 3.77 | Down |
| Nucleotides | 6-phosphogluconic acid | 1.57 | Down | 0.24 | Down | - | - |
| | cytidine-monophosphate degr prod | - | - | - | - | 2.27 | Down |
| | inosine 5'-monophosphate | 0.51 | Up | - | - | - | - |
| Sugar alcohols | Galactinol 1 | 0.59 | Up | - | - | 0.89 | Up |
| | myo-inositol | 0.86 | Down | 0.44 | Down | 0.60 | Down |
| | 2-Deoxyerythritol | - | - | - | - | 0.51 | Up |
| Glycoside | arbutin | - | - | - | - | - | - |
| Sugars | Glucose-1-phosphate | 0.62 | Down | 0.86 | Up | 0.99 | Up |
| | Sophorose 2 | 1.26 | Down | 0.87 | Up | 0.77 | Up |
| | D-Talose 2 | 0.40 | Up | 0.88 | Up | 1.17 | Down |
| | glucose 2 | 0.60 | Up | - | - | 0.70 | Up |
| | sorbose 2 | - | - | - | - | - | - |
| | fructose 1 | 0.48 | Up | 1.24 | Down | 1.27 | Down |
| | Gentiobiose 2 | 1.20 | Down | - | - | 0.89 | Up |
| | 2-Deoxy-D-galactose 1 | 1.45 | Down | 0.51 | Up | 0.41 | Up |
| | 2-deoxy-D-glucose 2 | 1.39 | Down | 0.75 | Up | 0.57 | Up |
| | Fructose 2,6-biphosphate degr prod 2 | 0.86 | Up | - | - | 0.88 | Up |
| | 1,5-Anhydroglucitol | 0.52 | Up | 1.26 | Down | - | - |
| | Erythrose 2 | - | - | - | - | 0.44 | Up |
| | mannose 1 | 0.27 | Up | - | - | 1.57 | Down |
| | beta-Mannosylglycerate 2 | 0.56 | Up | - | - | 0.65 | Up |
| | L-Threose 2 | 0.67 | Up | 1.30 | Down | - | - |
| | melibiose 2 | 0.77 | Up | 1.36 | Down | 1.31 | Down |
| | raffinose | 1.56 | Down | 0.67 | Up | - | - |
| | D-Talose 1 | - | - | 0.87 | Up | - | - |
| | Lyxose 2 | 1.24 | Down | - | - | - | - |
| | cellobiose 2 | - | - | - | - | - | - |
| | Digitoxose 2 | - | - | - | - | 1.52 | Down |
| | Threitol | - | - | 0.58 | Up | - | - |
| | sucrose | - | - | - | - | - | - |
| | maltitol | - | - | 1.49 | Down | 1.54 | Down |
| Glucuronic acids | Galactonic acid | 2.01 | Down | 0.34 | Up | 0.30 | Up |
| | D-galacturonic acid 2 | - | - | 0.65 | Up | 0.69 | Up |

| | | | | | | | |
|---|---|---|---|---|---|---|---|
| | mucic acid | 0.48 | Up | 0.58 | Up | - | - |
| | Saccharic acid | 0.66 | Up | 0.43 | Up | 0.94 | Up |
| | gluconic acid 1 | - | - | 0.40 | Up | - | - |
| | isocitric acid 2 | 0.96 | Up | 0.88 | Up | 1.16 | Down |
| | 3-Hydroxypropionic acid 1 | - | - | - | - | - | - |
| | Lactobionic Acid 1 | 0.77 | Up | 1.06 | Down | 0.83 | Up |
| | Itaconic acid | 0.25 | Up | 1.80 | Down | 0.54 | Up |
| | lactic acid | 2.10 | Down | - | - | 0.74 | Up |
| | Pyruvic acid | 0.62 | Down | 1.69 | Down | - | - |
| | 2-ketoadipate 3 | - | - | 1.30 | Down | - | - |
| | maleic acid | 0.36 | Up | - | - | 0.78 | Up |
| | succinic acid | 0.70 | Down | 0.68 | Up | 0.45 | Up |
| | fumaric acid | 0.46 | Down | 0.63 | Up | - | - |
| | 2-ketobutyric acid 2 | - | - | 1.48 | Down | 1.38 | Down |
| | glycolic acid | 0.81 | Up | - | - | 0.92 | Up |
| | salicylic acid | - | - | - | - | - | - |
| | oxalic acid | 1.36 | Down | - | - | - | - |
| | 2-keto-isovaleric acid 1 | - | - | 1.47 | Down | 1.24 | Down |
| | D-Glyceric acid | 0.68 | Up | - | - | 0.61 | Up |
| | tartaric acid | 0.44 | Down | 1.25 | Down | 1.64 | Down |
| | malonic acid 1 | - | - | 0.64 | Up | 0.76 | Up |
| | Indolelactate 2 | 0.37 | Up | 2.03 | Down | 1.92 | Down |
| Organic acids | 2-hydroxy-3-isopropylbutanedioic acid | 1.44 | Down | 0.47 | Up | 0.44 | Up |
| | citric acid | 0.86 | Up | 0.87 | Up | 1.14 | Down |
| | Citraconic acid degr1 | - | - | 1.59 | Down | 1.36 | Down |
| | Aconitic Acid | 0.59 | Up | 1.12 | Down | 0.73 | Up |
| | Glutaric Acid | 0.77 | Up | - | - | - | - |
| | L-Malic acid | - | - | 0.62 | Up | 0.82 | Up |
| | gentisic acid | 0.67 | Down | - | - | - | - |
| | benzoic acid | 0.44 | Down | 1.20 | Down | - | - |
| | Threonic acid | 1.24 | Down | 0.62 | Up | 0.48 | Up |
| | Tartronic acid | - | - | 1.41 | Down | 0.63 | Up |
| | 3-hydroxy-3-methylglutaric acid | 1.36 | Down | 0.55 | Up | 0.64 | Up |
| | Citramalic acid | - | - | - | - | - | - |
| | Dehydroascorbic Acid 1 | - | - | - | - | - | - |
| | Pelargonic acid | - | - | - | - | 0.71 | Up |
| | 4-Acetylbutyric acid 2 | - | - | - | - | 0.67 | Up |
| | 4-Hydroxybenzoic acid | - | - | - | - | - | - |
| | 2-methylfumarate | 0.49 | Down | 1.83 | Up | - | - |
| | 2-Furoic Acid | - | - | 0.94 | Down | 0.26 | Up |
| Fatty acid | palmitic acid | 0.62 | Up | 1.40 | Down | 1.98 | Down |
| | stearic acid | 0.50 | Up | 3.12 | Down | 1.73 | Down |

| | | | | | | | |
|---|---|---|---|---|---|---|---|
| | linoleic acid | - | - | 1.61 | Up | - | - |
| | 4-hydroxybutyrate | - | - | - | - | 0.43 | Up |
| Alcohols | 4-Methylbenzyl alcohol | - | - | 1.70 | Down | 1.44 | Down |
| | 1-Hexadecanol | 0.63 | Down | 2.40 | Down | 2.91 | Down |
| | xylitol | 1.34 | Down | 0.71 | Up | 0.57 | Up |
| | D-erythro-sphingosine 2 | 0.70 | Up | 1.73 | Down | 2.67 | Down |
| | sorbitol | 1.60 | Down | 0.34 | Up | 0.41 | Up |
| | mannitol | 0.75 | Up | 0.57 | Up | 0.87 | Up |
| | ribitol | 1.53 | Down | - | - | - | - |
| | Octadecanol | - | - | 1.77 | Down | - | - |
| | 1-octene-3-ol | - | - | 0.14 | Down | 0.56 | Down |
| | 1-amyl alcohol | - | - | - | - | - | - |
| Aldehyde | glutaraldehyde 4 | 0.70 | Up | 1.64 | Down | 1.41 | Down |
| | benzaldehyde | - | - | - | - | 2.85 | Down |
| | Hexanal | 1.86 | Up | 0.47 | Up | - | - |
| | enanthal | - | - | 0.44 | Down | 0.79 | Down |
| | 1-amyl alcohol | - | - | - | - | - | - |
| | E-2-hexenal | - | - | - | - | - | - |
| Ketone | prunin degr. Prod. 1 | - | - | 1.59 | Down | - | - |
| | 1,2-Cyclohexanedione 4 | - | - | 1.26 | Down | - | - |
| | 1,3-Cyclohexanedione 2 | 0.69 | Up | 0.72 | Down | 0.64 | Up |
| | 2,3-Butanedione | - | - | 1.34 | Down | 1.39 | Down |
| | 3-hydroxy-2-butanone | - | - | - | - | - | - |
| Esters | Dioctyl phthalate | 1.19 | Down | - | - | - | - |
| | Methyl Phosphate | - | - | - | - | - | - |
| | Diglycerol 1 | 1.35 | Down | 0.56 | Up | 0.61 | Up |
| | D-(glycerol 1-phosphate) | 0.84 | Up | - | - | 1.12 | Down |
| | Methyl benzoate | 0.42 | Down | 0.70 | Down | 0.18 | Down |
| Pyridins | 2-hydroxypyridine | - | - | 1.29 | Down | 1.24 | Down |
| Pyranones | 2-methoxy-3-isobutyl-pyrazine | - | - | 1.35 | Down | 1.19 | Down |
| Pyrazines | 4-Hydroxy-6-methyl-2-pyrone | 0.68 | Up | 2.03 | Down | 1.68 | Down |
| Lactones | Ribonic acid, gamma-lactone | 0.77 | Up | - | - | 0.61 | Up |
| | Gluconic lactone 2 | 1.45 | Down | 0.45 | Up | 0.41 | Up |
| | Gluconic lactone 1 | - | - | 0.55 | Up | 0.75 | Up |
| | D-erythronolactone 2 | - | - | - | - | 1.27 | Up |
| | γ-butyrolactone | 0.58 | Down | 0.57 | Up | - | - |
| Furan derivatives | 2-amylfuran | - | - | 0.37 | Down | - | - |
| Amines | Ethanolamine | - | - | 0.70 | Up | 0.47 | Up |
| | Tyramine | 1.33 | Up | 1.56 | Up | 1.74 | Up |
